# Strategies to increase the robustness of microbial cell factories

**DOI:** 10.1007/s44307-024-00018-8

**Published:** 2024-03-01

**Authors:** Pei Xu, Nuo-Qiao Lin, Zhi-Qian Zhang, Jian-Zhong Liu

**Affiliations:** 1 State Key Laboratory of Biocontrol, School of Life Sciences, Sun Yat-Sen University, Guangzhou, 510275 China; 2 Joint Research Center of Engineering Biology Technology of Sun Yat-Sen University and Tidetron Bioworks, Guangzhou, 510275 China; 3 Tidetron Bioworks Technology (Guangzhou) Co., Ltd., Guangzhou, 510399 China

**Keywords:** Host robustness, Regulatory factors, Cell membrane, Adaptive laboratory evolution, Computation biology

## Abstract

Engineering microbial cell factories have achieved much progress in producing fuels, natural products and bulk chemicals. However, in industrial fermentation, microbial cells often face various predictable and stochastic disturbances resulting from intermediate metabolites or end product toxicity, metabolic burden and harsh environment. These perturbances can potentially decrease productivity and titer. Therefore, strain robustness is essential to ensure reliable and sustainable production efficiency. In this review, the current strategies to improve host robustness were summarized, including knowledge-based engineering approaches, such as transcription factors, membrane/transporters and stress proteins, and the traditional adaptive laboratory evolution based on natural selection. Computation-assisted (e.g. GEMs, deep learning and machine learning) design of robust industrial hosts was also introduced. Furthermore, the challenges and future perspectives on engineering microbial host robustness are proposed to promote the development of green, efficient and sustainable biomanufacturers.

## Introduction

Engineering microbial cell factories have been widely applied to produce various chemicals, such as natural products, biofuels, and bulk chemicals (Cho et al. 2022; Zhu et al. 2023). Metabolic engineering and synthetic biology enable the design of kinds of advanced cell factories mostly by introducing heterologous or non-natural biosynthetic pathways into host strains. From the previous complete biosynthesis of opioids (Galanie et al. 2015) to the de novo biosynthesis of xanthohumol (Yang et al. 2024), yeasts have shown great potential in the biosynthesis of many high-value active compounds. In addition to the model strains such as *Escherichia coli* and *Saccharomyces cerevisiae*, several microorganisms have been engineered as important chassis cells to adapt different application environments, such as *Zymomonas mobilis* (Wang et al. 2018), *Yarrowia lipolytica* (Park and Ledesma-Amaro 2023), and *Halomonas campaniensis* (Ling et al. 2019), with the aid of powerful genome-editing tools. A series of strategies based on metabolic engineering and systematic biology have been developed to improve the productivity of microbial cell factories, mainly by fine-tuning heterologous pathways (Chen et al. 2022; Ding and Liu 2023; Yan et al. 2023), eliminating the rate-limiting enzymatic steps (Li et al. 2020) and host engineering to block competing pathways (Ma et al. 2019).

Despite the great progress achieved by these strategies, engineering microbial cells to meet industrial requirements remains a challenge. In the large-scale fermentation process, microbial cells constantly face perturbations resulting from genetic and phenotypic instability, metabolic imbalance, and various harsh industrial conditions (including low pH, high temperature, and metabolite toxicity), which lead to poorly performing strains under these conditions. However, engineered microbial cells in the laboratory often do not take into account these multiple disturbances encountered in industrial conditions. Microbial robustness refers to the ability of the microbe to maintain constant production performance (defined as titers, yields, and productivity) regardless of the various stochastic and predictable perturbations that occur in a scale-up bioprocess (Mohedano et al. 2022; Olsson et al. 2022). Poor robustness limits industrial-scale microbial production.

The concept of microbial robustness goes beyond that of tolerance, even though they have sometimes been used interchangeably in industrial microbial applications. Tolerance or resistance refers to the ability of cells to grow or survive when exposed to single or multiple perturbations. It is generally described only in terms of growth-related parameters (such as viability or specific growth rate). Robustness represents the ability of a strain to maintain a stable production performance (e.g. titer, yield, and productivity) when growth conditions are changed. Strains with higher tolerance do not guarantee a higher yield, while the strain with higher robustness must have a higher tolerance. Therefore, increasing the strain robustness against unfavorable conditions becomes one of the most important considerations in engineering microbial cell factories and extending them to practical applications.

**Fig. 1:**
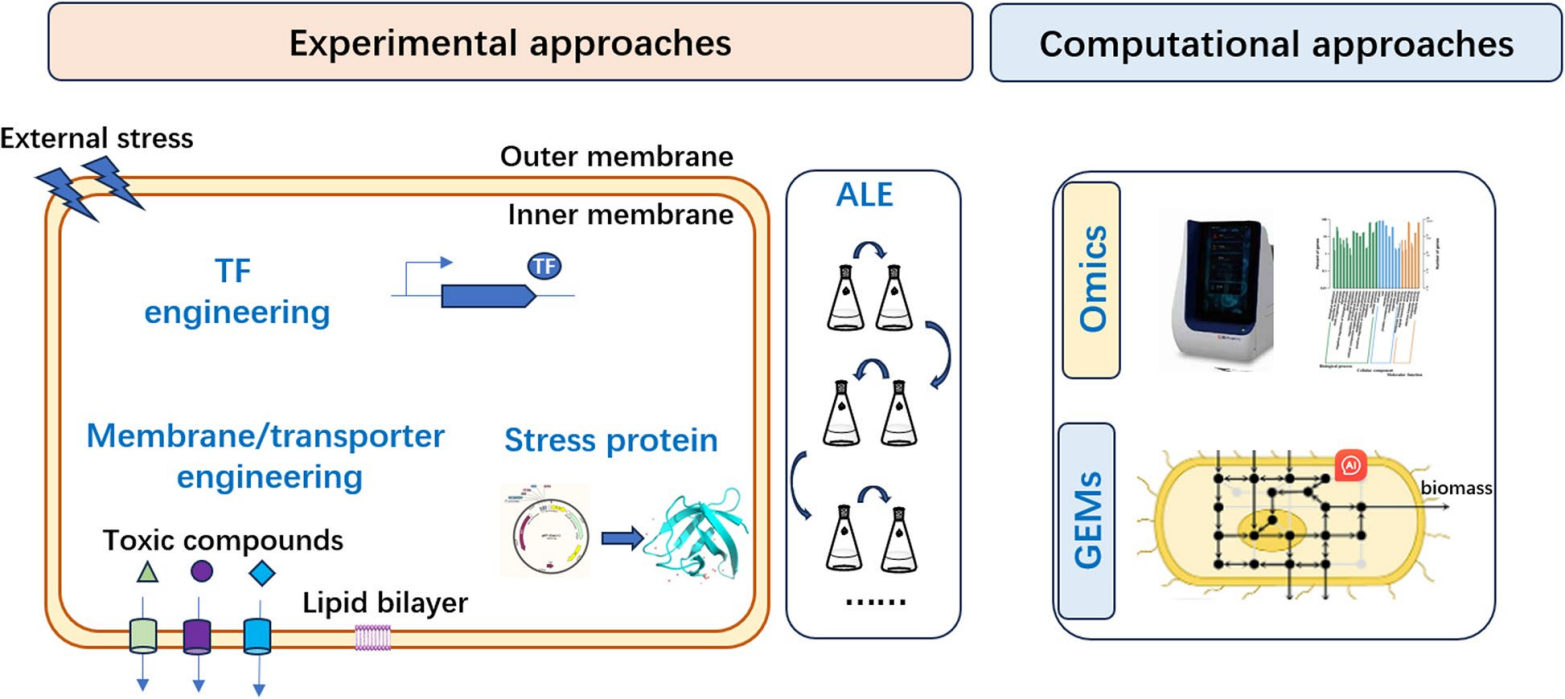
Strategies for engineering robust microbial cell factory

In this review, we focus on the introduction of the most proven strategies in engineering microbial robustness for high titer and productivity (Fig. 1). In addition, the challenges and future perspectives of microbial host engineering for increased robustness are discussed.

### Transcription factor engineering

Transcription factors (TFs) are key proteins that control the fine-tuning expression of target genes by activating or suppressing gene transcription in a variety of biological processes (He et al. 2023). Cells have evolved to optimize cellular function through the coordinated regulation of multiple enzymes and pathways by different transcription factors in response to different environmental conditions. Based on their regulatory scope, transcription factors can be divided into global and specific transcription factors (Yu and Gerstein 2006). Global transcription factors can initiate or repress the expression of different genes involved in different physiological activities. The seven most well-characterized global regulatory factors, including CRP, IHF, FNR, ArcA, FIS, Lrp, and NarL, control over 50% of the *E. coli* genes (Lin et al. 2013). In a pyramidal gene expression network of *E. coli*, the top global regulatory factors control the middle high-level regulatory factors, which further regulate the low-level regulatory factors. Through a hierarchical regulation, the transcription and expression of target genes are systematically controlled in the genome-wide metabolic network. Therefore, the transcription factor has become a feasible and efficient target for improving strain robustness (Table 1).

**Table 1:** Strategies for transcriptional factor engineering

Gene	Host	Strategy	Tolerance	Robustness	Reference
*rpoD*	*E. coli*	gTME	Ethanol-tolerance	No detection	Alper and Stephanopoulos 2007
*rpoD*	*Klebsiella pneumoniae*	gTME	Xylose	2,3-butanediol production increased by 228.5%	Guo et al. 2018
*Rpb7*	*S. cerevisiae*	gTME	10% ethanol	40% increase in ethanol titers	Qiu and Jiang 2017
*sigA*	*R. ruber*	gTME	40% acrylamide	No detection	Ma and Yu 2012
*rpoD*	*Z. mobili*	gTME	9% ethanol	Two-fold increase in ethanol production	Tan et al. 2016a
*crp*	*E. coli*	Overexpression of the *crp* mutant (K52I/K130E)	0.9 mol/L NaCl	No detection	Zhang et al. 2012
*crp*	*E. coli*	Overexpression of the *crp* mutant (S179P/H199R)	1.2%(v/v) isobutanol	No detection	Chong et al. 2014
*irrE*	*E. coli*	Overexpression of *Deinococcus radiodurans irrE* mutant	Ethanol, butanol	No detection	Chen et al. 2011
*DR1558*	*E. coli*	Overexpression of *D. radiodurans irrE* mutant	300 g/L glucose and 2 M NaCl	No detection	Guo et al. 2017
*FucO*	*E. coli*	Overexpression of the fucO mutant (L7F)	Furfural	No detection	Zheng et al. 2013
*RamA*	*C. glutamicum*	Overexpression	No detection	Improve N-acetylglucosamine production	Deng et al. 2021
*Haa1*	*S. cerevisiae*	Overexpression Haa1S135F	Acetic acid	No detection	Swinnen et al. 2017
Artificial transcription factor	*E. coli*	Overexpression	Heat, osmotic pressure and cold shock	Improved resistance	Lee et al. 2008

Global transcription machinery engineering (gTME), which focuses on introducing mutations in generic transcription-related proteins that trigger the reprogramming of gene networks and cellular metabolism, has proven to be a versatile approach to altering cell robustness. For example, engineering the housekeeping sigma factor δ^70^ improved the *E. coli* tolerance to 60 g/L ethanol and high concentrations of SDS, while resulting in a high yield of lycopene (Alper and Stephanopoulos 2007). The gTME strategy has also been used in the more complex eukaryotic transcriptional machinery *S. cerevisiae* to increase its resistance to high concentrations of glucose and ethanol. Two target proteins Spt15 and Taf25 were selected for constructing ep-PCR gene libraries, and the resulting best mutant spt15-300 showed a significant growth improvement in the presence of 6% (v/v) ethanol and 100 g/L glucose (Alper et al. 2006). Further studies extended the gTME method to different organisms such as *Lactobacillus plantarum, Rhodococcus ruber,* and* Z. mobilis* to enhance their acid tolerance, acrylamide tolerance, and ethanol tolerance, respectively (Klein-Marcuschamer and Stephanopoulos 2008; Ma and Yu 2012; Tan et al. 2016a).

In addition to δ^70^, the cAMP receptor protein (CRP), which regulates more than 400 genes, has been successfully evolved to improve alcohol tolerance, and acid tolerance, and increase biosynthetic capacities such as vanillin, naringenin and caffeic acid (Basak et al. 2014; Geng and Jiang 2015; Zhang et al. 2023). For example, heterologous expression of the global regulator *irrE* from *Deinococcus radiodurans* and its mutant IrrE increased tolerance against ethanol or butanol stress in *E. coli* by 10 to 100-fold (Chen et al. 2011). Thereafter, by overexpression of the response regulator DR1558 from *D. radiodurans,* the engineered *E. coli* increased tolerance to osmotic stress at high concentrations of 300 g/L glucose and 2 mol/L NaCl (Guo et al. 2017). GlxR, the CRP homolog in *Corynebacterium glutamicum*, can directly control the transcription of approximately 14% of annotated genes (Kohl and Tauch 2009). Overexpression of GlxR and the other global transcription factors RamA and SugR significantly improves the N-acetylglucosamine biosynthesis in *C. glutamicum* (Deng et al. 2021)*.* Zinc finger-based artificial transcription factors have also been developed to modulate gene expression in various organisms (Lee et al. 2008; Negi et al. 2023).

Unlike the global transcription factors, specific transcription factors generally regulate the individual gene expression, including transcriptional activators and repressors. The regulon-specific transcription factor Haa1, which is involved in the activation of approximately 80% of the acetic acid-responsive genes, has been engineered to improve acetic acid tolerance in *S. cerevisiae* (Cunha et al. 2018; Swinnen et al. 2017).

As mentioned above, the diversity of transcription factors from different organisms provides more opportunities to engineer microbial cell factories with different resistance to different scale-up conditions. For example, for a specific tolerance such as ethanol, different transcription factors (e.g. rpoD, Rpb7 and irrE) could be selected for engineering by overexpression of their wild-type or mutant. At the same time, the TF such as Crp could be used to enhance multiple tolerance while maintaining the production capacity of the host cells. From the viewpoint of metabolic engineering, transcription factors have the unique merit of providing “multi-point regulation” to compensate for the insufficient effect of single-key gene modification.

### Membrane/transporter engineering

The cell membrane outlines the cell border between the cell itself and the surrounding environment, and mediates the energy exchange, transportation of metabolites and extracellular communication (Coskun and Simons 2011). Described as a dynamic bilayer, the cell membrane is composed of different types of lipids, carbohydrates and proteins. The lipid bilayer, composed mainly of phospholipids, acts as an important physical barrier against osmotic pressure, the (bio)chemical environment and mechanical stress. In an industrial process, cells often face membrane damage caused by the accumulation of metabolites and acidic toxicity. Maintaining the function of cell membranes is a feasible and efficient way to improve the tolerance and productivity of industrial microbes (Table 2).

**Table 2:** Strategies for membrane/transporter engineering

Gene	Host	Strategy	Tolerance	Robustness	Reference
*ttgB*	*E. coli*	Overexpression	α-Pinene	Increased cell growth	Dunlop et al. 2011
*Yp_692684*	*E. coli*	Overexpression	Geranyl acetate	Increased Limonene yield	Dunlop et al. 2011
*mexF*	*E. coli*	Overexpression	Farnesyl hexanoate	Increased cell growth	Dunlop et al. 2011
*MaeI*	*S. cerevisiae*	Overexpression	Acids	Increased yield of succinate, maleic acid, and fumaric acid	Darbani et al. 2019
*cis*–trans isomerase (Cti) gene	*E. coli* MG1655	Incorporated trans-unsaturated fatty acids	Low pH, high temperature	Improved production of carboxylic acids, styrene	Tan et al. 2016b
Δ9 desaturase Ole1 gene	*S. cerevisiae*	Improved membrane oleic acid content	Acids, NaCl and ethanol	No detection	Nasution et al. 2017
acyl-ACP thioesterase gene	*E. coli*	Reduced unsaturated fatty acid content	Free fatty acids	Improved cell viability	Lennen and Pfleger 2013
*Acc1*^*S1157A*^	*S. cerevisiae*	Overexpression, increased oleic acid content	*n*-Butanol, 2-propanol, and hexanoic acid	Increased cell viability at C8 stress	Besada-Lombana et al. 2017
*AcrAB-TolC*	*E. coli*	Overexpression	Styrene and 1-hexene	Improved styrene production to about 17 mg L − 1	Mingardon et al. 2015
*ompA* and *fadL*	*E. coli*	Overexpression	Phenylpropanoids, resveratrol, naringenin and rutin	Increased tolerance	Zhou et al. 2015
*Erg*	*S. cerevisiae*	Mutation	Heat	Improved cell growth and ethanol production at > 40 °C	Caspeta et al. 2014
*Erg*	*S. cerevisiae*	Deletion	Heat	improved cell growth at 39 °C	Liu et al. 2017
*CDS1* and CHO1	*S. cerevisiae*	Overexpression	NaCl	No detection	Yin et al. 2020
*pssA*	*E. coli*	Overexpression	Octanoic acid, toluene, ethanol	Increased production of biorenewables	Tan et al. 2017
*tolC*	*E. coli*	Overexpression of *tolC* together with ABC family transporters or MFS family transporters	No detection	Increased amorphadiene titer by more than threefold	Zhang et al. 2016

Membrane engineering can be implemented by improving the integrity, regulating the mobility and controlling the permeability of the membrane, mainly by engineering the fatty acid composition and lipid composition. The former strategy usually focused on the alteration of lipid saturation, average chain length and integration of cyclopropane fatty acids. For example, the transcription of two essential genes *fabA* and *fabB* regulated by a two-component system CpxRA could boost the biosynthesis of unsaturated fatty acids (UFAs) to enhance the UFAs content in membrane lipids (Xu et al. 2020). This mechanism enables *E. coli* to grow at pH 4.2, and also works in *Salmonella* Typhimurium LT2 and *Shigella flexneri* 2a str. 2457T due to their high FabA identity with *E. coli* FabA. Overexpression of the Δ9 desaturase Ole1 from *S. cerevisiae* increased the ratio of unsaturated to saturated fatty acids by increasing the membrane oleic acid content, thereby improving the tolerance to various stresses, such as acid type, NaCl and ethanol (Nasution et al. 2017). In a previous work, the rat elongase 2 gene (rELO2) was overexpressed in *S. cerevisiae* and a strain tolerant to ethanol, *n*-propanol and *n*-butanol was obtained due to its increased intracellular oleic acid content (Yazawa et al. 2011). By the overexpression of the cis–trans isomerase (Cti) from *Pseudomonas aeruginosa*, the cell membrane of *E. coli* MG1655 was incorporated with trans-unsaturated fatty acids, leading to lower membrane fluidity and enhanced tolerance to carboxylic acids, styrene and butanol (Tan et al. 2016b). Compared to saturated acids, unsaturated fatty acids usually have a lower melting point, resulting in more fluidity within the lipid bilayer (Hassan et al. 2020). In another work, to enhance the membrane integrity and cell viability of a free fatty acid-producing *E. coli*, a *Geobacillus* acyl-ACP thioesterase was used to reduce the unsaturated fatty acid content in the membrane to avoid the cell lysis (Lennen and Pfleger 2013).

Changing the lipid composition is another effective approach to membrane engineering, such as altering sterol content, lipid length and phospholipid head groups. In *S. cerevisiae*, the substitution of ergosterol with fecosterol by an ERG3 mutant coupled with upregulation of sterol synthesis improved cell growth and ethanol production at temperatures above 40 °C (Caspeta et al. 2014). By single or combined deletion of different *erg* genes (e.g. *Δerg2, Δerg3, Δerg4, Δerg5* and *Δerg3Δerg5*), the engineered strain achieved up to 2.24-fold higher growth rate than the wild-type strain at 39 °C (Liu et al. 2017). These results highlight the significant role of sterols in the resistance of yeast to exogenous stresses, and indicate the possibility of increasing the yeast robustness by engineering their sterol composition. Recently, the co-expression of two genes involved in the phospholipid synthesis pathway, *CDS1* and *CHO1*, improved the salt stress tolerance of *S. cerevisiae* up to 1.2 mol/L NaCl (Yin et al. 2020). Interestingly, overexpression of *ELO2* in *S. cerevisiae* also improved its tolerance to osmotic stress, benefitting from the enhanced membrane integrity caused by the increase in the long fatty acid content of sphingolipids (Zhu et al. 2020a). In the fatty acid production process, deletion of the *aas* gene (encoding acyl-ACP synthase) decreased the incorporation of FFAs into membrane phospholipids and increased fatty acid production (Sherkhanov et al. 2014). In another work, the phospholipid head was engineered by increasing the expression of phosphatidylserine synthase (pssA) to increase the tolerance of *E. coli* to octanoic acid, which is a membrane-damaging solvent. At the same time, tolerance to other industrially relevant inhibitors, such as toluene, ethanol, furfural, and low pH was also improved (Tan et al. 2017). This work suggests that engineering the lipid composition can change the membrane integrity, hydrophobicity and fluidity effectively, thus effectively reducing the penetration of toxic compounds into the cell and consequent cell damage.

Many cell transporter proteins play various important roles in physiological activities such as nutrient uptake, metabolite release and cell signaling. Transporter engineering facilitates the transmembrane transport of matrix, intermediate metabolites, and end products in microbial cells, thereby alleviating feedback inhibition and cytotoxicity of intermediate metabolites and end products. Transporter proteins usually consist of influx and efflux proteins, while the latter is mainly the target for engineering cell robustness. A large number of membrane proteins have been demonstrated to be involved in the efflux of a series of compounds (Jiang et al. 2021b; Yamada et al. 2021; Zhu et al. 2020b). Overexpressing these efflux pumps successfully improves microbial tolerance and robustness (Table 2). For example, Dunlop et al. (2011) expressed 43 efflux pumps homologous to *Pseudomonas putinosa* protein in *E. coli*, improving tolerance to a variety of terpenes, biofuels such as n-butanol and isoamyl alcohol. However, these efflux pumps often consume ATP or ion gradients as an energy source for the transport of matter. Recently, it was found that overexpression of the voltage-dependent transport protein SpMae1 from *Schizomyces cerevisiae* in *S. cerevisiae* can increase the yield of succinate, maleic acid, and fumaric acid by a factor of 3, 8 and 5 times, respectively, without affecting the growth of *S. cerevisiae*, and the transport protein SpMae1 does not need to consume ATP and Na^+^ as transport energy (Darbani et al. 2019). These results suggest that there is great potential to apply efflux proteins from unrelated organisms in chassis cells to exhibit their native advantage.

Despite the potential benefits of transporter engineering in improving cell robustness, this method has not been systematically explored in strain construction, mainly due to the difficulties in transporter discovery, characterization and manipulation. On the other hand, due to their similar structures and broad substrate promiscuity, a transporter can efflux the intermediate metabolites and reduce the yield of the final product, especially in the context of natural product biosynthesis.

### Stress protein engineering

Microbes used in industrial bioprocess applications often encounter multiple stresses that negatively affect cell growth and productivity. Stress resistance is therefore important to guarantee the productive robustness of the cells (Table 3). Several stress proteins have been used to improve the tolerance and robustness of microorganisms. Twelve heat shock proteins (HSPs) from the thermophiles *Geobacillus* and *Parageobacillus* were expressed in the riboflavin-producing *B. subtilis* 446, ten of which improved the heat resistance of strains (Wang et al. 2019).

**Table 3:** Strategies for stress protein engineering

Gene	Host	Strategy	Tolerance	Robustness	Reference
*hsp20-1/2/3/4/5, PtdnaK, PtgroEL, PtdnaJ, PtgrpE, GtgrpE, Pthsp33, PtgroES*	*B. subtilis*	Overexpression of the *HSP* genes from thermophiles *Geobacillus* and *Parageobacillus*	Heat + 10% NaCl	Improved riboflavin titers at higher fermentation temperatures and shortened fermentation period	Wang et al. 2019
*groES, thiF*	*E. coli*	IMHeRE	Heat	Improved lysine production up to 5-fold	Jia et al. 2016
*cspL*	*E. coli* and *S. cerevisiae*	Overexpression of *B. coagulans* 2–6 *cspL*	Heat	Enhanced high-temperature growth	Zhou et al. 2021
*dsrA, hfq*	*E. coli*	Overexpression of the small noncoding RNA (sRNA) DsrA t Hfq	Acid (pH4.5)	Improved growth	Lin et al. 2021
*hu, rbp* and *clpP*	*E. coli*	Overexpression of synthetic operons with novel genes from extreme environment	Acid (pH1.9)	Increased 100-fold of survival percentage of cells	de Siqueira et al. 2020
*SOD2-TTHA1300*	*S. cerevisiae*	*artificial antioxidant defense system*	Heat	Improved ethanol production	Xu et al. 2018
*SOD1, GSH1 GLR1*	*S. cerevisiae*	Dynamic feedback regulation of the anti-stress genetic circuits	Heat and ROS	Improved ethanol production	Qin et al. 2020
*katE, katG, sodA, sodB, sodC*	*E. coli*	Overexpression of CAT and SOD	5-Aminolevulinic acid	Improved 5-aminolevulinic acid production	Zhu et al. 2019
*otsA, otsB, treS*	*E. coli*	Overexpression of *Arthrobacter simplex* trehalose biosynthesis genes	4% ethanol, 5% methanol, 0.6 M NaCl, 0.025% H_2_O_2_	Improved growth	Cheng et al. 2020

Interestingly, three HSPs with the best heat resistance could improve the ability of the strain to resist osmotic stress of 10% NaCl, and also promoted the synthesis of riboflavin, i.e. improved the robustness of the strain. Li’s group screened a series of HSP elements and constructed an intelligent microbial heat-regulating engine (IMHeRE), which contains a thermotolerant system and a quorum sensing system. Using the IMHeRE gene circuit, the optimal growth temperature range of *E. coli* was broadened, allowing a 5-fold increase in the production of lysine at 40 °C (Jia et al. 2016). In addition to HSPs, a cold shock protein (CspL), an RNA chaperone from a lactate-producing thermophile *B. coagulans* 2–6, could also confer strong high-temperature resistance to mesophilic industrial strains (Zhou et al. 2021). The protein not only enhanced the growth ability of *E. coli* and *S. cerevisiae* at high temperatures (45 °C and 36 °C, respectively), but also promoted the cell growth rates at normal temperatures. In a practical fermentation, high temperature can not only hinder the competing microorganisms contamination and invading bacteriophages, but also improve the substrate saccharification efficiency.

Acid tolerance is another desirable phenotype for many industrial microorganisms. Simultaneous overexpression of the non-coding small RNA (sRNA) DsrA and its chaperone Hfq in *E. coli* significantly improved its tolerance under a moderately acidic conditions (pH 4.5) (Lin et al. 2021). In addition to simply expressing a single resistance gene, it is also possible to engineer microorganisms with different levels of stress tolerance by combining different stress proteins. For example, by combining three synthetic ribosome binding sites and three acid resistance genes, *hu*, *rbp* and *clpP*, the engineered *E. coli* was able to achieve a survival rate more than 100 times higher than the wild type under an acidic shock at pH 1.9 (de Siqueira et al. 2020). This work showed that there is a non-linear relationship between observed cell survival rates and predicted protein expression, since increasing RBS strength did not always lead to increased acid resistance.

During a normal growth metabolism, cells constantly produce reactive oxygen radicals (ROS) that lead to cell damage or death. In the process of large-scale industrial fermentation, a variety of stress factors will accelerate the accumulation of ROS and exacerbate cell damage. To overcome the detrimental effects of oxidative stress on yeast in a high-temperature fermentation, an artificial antioxidant defense system was constructed by mining fifteen antioxidant genes from *Thermus thermophiles* HB8 and *S. cerevisiae*, and integrating them into the *S. cerevisiae* genome respectively (Xu et al. 2018). The engineered strains showed improved heat tolerance, increased metabolic capacity (growth rate and cell viability) and higher ethanol yield. They also enhanced the strain’s tolerance to ROS by strengthening the glutathione synthesis pathway, thereby improving the robustness and ethanol production of *S. cerevisiae* (Qin et al. 2020). A similar strategy also works in *E. coli*. Overexpression of CAT and SOD in *E. coli* not only improved its tolerance to 5-aminolevulinic acid, but also promoted the synthesis of 5-aminolevulinic acid (Zhu et al. 2019). Simultaneous expression of *katE* and *sodB* resulted in an 81% and 117% increase in biomass and 5-aminolevulinic acid yield, respectively.

High concentrations of substrates and metabolites (e.g., acetate, end products, etc.) can lead to high osmotic pressure, loss of intracellular water, and cytoplasmic dehydration. To compensate for high osmotic pressure, microorganisms often maintain intracellular water by synthesizing large amounts of endogenous osmo-protectants such as trehalose. Overexpression of the trehalose biosynthetic genes (*otsA*, *otsB*, and *treS*) from *Arthrobacter simplex* in *E. coli* can induce the cell to synthesize trehalose as an osmo-protector, thereby greatly improving its tolerance to ethanol (Cheng et al. 2020).

Similar to TFs, a few stress proteins are multi functional to resist different external stress. In a practical application, engineering a certain stress protein (e.g. GroES) or synergistically utilizing various stress proteins might enable the host to tolerate different stresses synchronously, such as heat and osmotic pressure.

### Adaptive laboratory evolution

Adaptive Laboratory Evolution (ALE) strategies rely on the natural mutations that occur in microorganisms under taming pressures such as pH, temperature or toxin concentration. Target mutants with expected tolerance can be produced by continuous culture over hundreds of generations under specific conditions. ALE has been used with great success to improve the robustness of microbial cells, thereby reducing costs in industrial applications (Table 4).

**Table 4:** Strategies for adaptive laboratory evolution

Gene	Host	Strategy	Tolerance	Robustness	Reference
*ESBP6*	*S. cerevisiae*	Overexpression of *ESBP6*	Acid (pH3.5), aromatic acids (coumaric acid, ferulic acid)	Improved coumaric acid production	Pereira et al. 2020
*rpoC*^*H419P*^*,*	*E. coli*	Overexpression of *rpoC*^*H419P*^	Octanoic acid (C8)	Improved octanoic acid (C8) production	Chen et al. 2020
*rnb, envZ, recC*	*E. coli*	ALE	Large-scale culture conditions	Improved astaxanthin production	Lu et al. 2022
*RpoA*^*S266P*^	*E. coli*	ALE	85 mM acetate	Improved growth	Rajaraman et al. 2016

Combined with omics analysis, one can deeply understand the tolerance mechanism to selection pressure and establish the relationship between the phenotype and genotype. In a typical process, ALE is firstly used to obtain tolerant microorganisms, and then resequencing and omics analysis of functional genomes can be used to explore the robustness-related gene elements. Finally, knockout, overexpression or activation/inhibition of the target genes is used to determine the effect of the target gene on the tolerance or titer. In a recent study, ALE was performed on *S. cerevisiae* at low pH (3.5) in the presence of *p*-coumaric acid and ferulic acid, resulting in mutants tolerant to both aromatic acids and low pH conditions (Pereira et al. 2020). Resequencing and functional genomic analysis confirmed that the transport protein Esbp6 was the key factor responsible for the increased tolerance. Overexpression of the *ESBP6* gene could not only improve its tolerance, but also promote the synthesis of aromatic compounds, i.e. improve robustness. Another study found that the mutant RpoC^H419P^ of the RNA polymerase subunit RpoC in an evolved *E. coli* was associated with its increased tolerance to octanoic acid (C8) and increased C8 production (Chen et al. 2020). Using ALE coupled with genome restructuring technology, we obtained a robust astaxanthin-producing *E. coli* that was tolerant to large-scale culture high-density fermentation conditions (low pH, high osmolality (high NaCl concentration), high acetate concentration, and high reactive oxygen species concentration (high concentration of H_2_O_2_)), and found that *rnb*, *envZ*, and *recC* were associated with the robustness of the strain (Lu et al. 2022).

In the sequencing era, omics-based analysis becomes pivotal for ALE to discover functional genes. The unbiased high-throughput genome-scale screens can accelerate this analysis process. Several important technologies have emerged to study the genotype–phenotype associations related to robustness. Transposon sequencing (Tn-seq) is becoming an efficient functional genomics technology based on transposon mutagenesis and next-generation sequencing (NGS) (Kwon et al. 2016). This approach has several advantages, such as fast and efficient processing capability, wide coverage, and high accuracy, and has been utilized in a variety of bacterial species to provide comprehensive information on gene functions. Integration of RNA-sequencing and RNA interference (RNAi) is also proved to be an efficient method to investigate the molecular mechanisms responsible for microbial tolerance (Zheng et al. 2022). Clustered regularly interspaced short palindromic repeats (CRISPR) screens, which utilize the efficiency and flexibility of CRISPR-Cas (CRISPR-associated protein) genome editing, have become another popular and productive tool for uncovering previously unknown molecular mechanisms (Doench 2018; Bock et al. 2022). As a typical gene repression tool, CRISPR interference (CRISPRi) enables genome-scale analysis of gene function based on pooled and arrayed guide RNA (gRNA) libraries. This approach has been successfully implemented in a series of microorganisms with medical and industrial significance (Sun et al. 2023). In the case of CRISPRi screening of *S. cerevisiae* tolerance against acetic acid stress, an arrayed library consisted of > 9000 strains with > 18,000 gRNAs targeting > 1000 essential genes was constructed, and several respiratory growth-essential genes were identified to be involved in acetic acid tolerance regulation (Mukherjee et al. 2021).

Although ALE is a powerful approach for mutant screening, there are several aspects that need to consider. Typically, the subsequent multi-omics sequencing and analysis is time-consuming and costly, especially for high-throughput experiments. Sometimes it is challenging to focus on the effect factor among the discovered key genes. A publicly available database ALEdb (ALEdb.org) has been assembled by a collection of the mutations acquired during ALE (Phaneuf et al. 2019), which contains 738,051 instances of 229,062 unique mutations from 11,841 isolates across 232 unique projects, thus helping researchers to rationally design engineered *E. coli* mutants (Catoiu et al. 2023). In addition, the evolved tolerant strain may show unexpectedly low production titers, rate or yield.

### Computation-assisted robustness design

The aforementioned experimental methods can, to a certain extent, tune the performance of microbial cells to resist harsh industrial conditions. However, traditional regulatory strategies generally require a continuous design-build-test-learn cycle, which is time-consuming and laborious. More importantly, the intrinsic regulatory mechanism is complex. For example, the transporter protein is not always specific for certain compounds. Broad substrate specificity increases the uncertainty.

Genome-scale models (GEMs) have developed as one computational system biology approach to interpret and integrate multi-omics data. GEMs can be used to compute the metabolic and proteomic state of a microorganisms. Many GEMs have been constructed for typical industrial microorganisms, such as *E. coli* (Mao et al. 2022), *S. cerevisiae* (Lu et al. 2021), and *B. subtilis* (Kocabaş et al. 2017). Due to the biological complexity, such GEMs are generally integrated with different constraints to predict phenotype from genotype more accurately. As for *E. coli*, three stress-specific GEMs, FoldME (Chen et al. 2017), OxidizeME (Yang et al. 2019) and AcidifyME (Du et al. 2019), have been constructed for various environmental pressures. FoldME, a thermal-stress-response model, delineates the in vivo protein folding through the competition between de novo spontaneous folding and chaperone-mediated (HSP70 or HSP60) folding pathways. OxidizeME, a ROS-stress-response model, computes the systems-level balance between ROS management and iron homeostasis, including demetallation/mismetallation of Fe(II) proteins, damage and repair of iron–sulfur clusters and DNA damage. AcidifyME, an acid-stress-response model, established a quantitative framework integrated with characterized acid resistance mechanisms, including membrane lipid fatty acid composition, pH-dependent periplasmic or membrane protein activity and stability, and periplasmic chaperone protection. Such GEMs enable the rational and fast design of host robustness from a computational viewpoint.

With the help of mathematical models such as machine learning or deep learning, the performance of cell robustness may be adjusted quickly and accurately without taking into account the complex mechanism of action. Deep learning is an algorithm that uses artificial neural networks (for example, convolutional neural networks (CNNS) and recurrent neural networks (recurrent neural networks). RNN)) as a framework for characterizing and learning data sets (Sapoval et al. 2022). Machine learning uses algorithms such as Bayes, support vector machine and logistic regression to uncover the hidden rules and essence behind things, and to obtain models through training data sets (Asnicar et al. 2023). By developing machine learning or deep learning models, any biological sequence such as DNA, RNA or amino acid sequence can be used as data input to solve many biological problems. For example, by combining machine learning with abundant proteomics and metabolomics data, the pathway dynamics can be effectively predicted in an automated manner (Costello and Martin 2018). This approach outperforms the classical kinetic models, which rely heavily on domain expertise, and guides the bioengineering efforts with qualitative and quantitative predictive data. Additionally, introducing machine learning or deep learning into multi-scale GEMs can effectively improve the model quality and prediction accuracy.

## Conclusion and future perspectives

A stable microbial cell is more economically feasible to scale up from the laboratory testing to industrial biomanufacturing. In this review, we summarized the current strategies to improve host robustness, including three knowledge-guided engineering approaches such as transcription factors, membrane/transporter and stress proteins, and adaptive laboratory evolution based on natural selection. In addition, artificial intelligence (e.g. deep learning and machine learning)-assisted pathway design shows great potential in the design of robust industrial hosts. The above strategies have effectively improved the robustness of microbial hosts and expanded their applications in biomanufacturing. However, there are still several challenges in engineering cell robustness.

First, the understanding of the mechanisms of toxicity and robustness is limited. Although the transcription factor engineering allows the regulation of the entire metabolic network, the diversity makes it difficult to focus on which factor to engineer. In most cases, a trial-and-error approach is used to screen for the most effective factors. It is therefore expected that rapid and easy-to-engineer methods will be developed for mining and modifying regulatory factors, thereby promoting the high-throughput and (semi-)rational construction of microbial cell factories. Meanwhile, cell metabolism can be manipulated by combining multiple transcription factors to control a variety of key proteins to different harsh conditions at the same time. For example, a method called MultIplex Navigation of Global Regulatory Networks (MINR) has been proposed to target multiple transcription factors simultaneously (Liu et al. 2019). Based on these experimental data, the distinct regulatory mechanism for each known transcription factor can be uncovered to build a model or database. Alternatively, the functions of most transporters are unknown. Similar to transcription factors, the identification and characterization of transporters for specific compounds with high efficiency is also required.

Second, ALE is an efficient tool for engineering microbial cells with specific phenotypes, whereas the isolation of the target mutant from a microflora usually requies a high-throughput facility. For example, the DREM CELL platform allows for the screening of target strains at a picoliter scale (Meng et al. 2022). Depending on the fluorescence output, a biosensor based on transcription factors or riboswitches can significantly increase the efficiency of a screening process (Li et al. 2023). In addition, biosensors with appropriate sensitivity and dynamic range can be used to dynamically regulate the biosynthesis of many compounds (Hossain et al. 2020). A robust biosensor may be based on existing ones or modified to facilitate the construction of robust cell factories.

Third, model microorganisms, such as *E. coli* and *S. cerevisiae*, are usually mesophilic and have limited ability to withstand harsh industrial stresses. For example, C1 biotechnology has made great progress in using CO_2_, methanol, formic acid, etc. to synthesize valuable compounds in model hosts (Bae et al. 2022; Zhan et al. 2023). To improve the host’s robustness to cope with these substrates, the reconstruction of metabolic pathways to reduce the toxicity of substrates or intermediates is the necessary step. In another case of non-model host *Halomonas bluephagenesis*, an important platform chemical 3-hydroxypropionic acid, achieved high yields ofup to 154 g/ L at a 60 g/L of NaCl (Jiang et al. 2021a), which is intolerable for model hosts. Recently, knowledge of genome editing tools has increased, making it easier to work with non-model hosts (Liu et al. 2022). Some non-model hosts, such as thermophilic and acidophilic strains, may become an important direction for future cell factory construction (Thorwall et al. 2020), which can address the limitations of model microorganisms.

The rapid development of machine learning and deep learning has led to the emergence of many biological tools with various functions, such as DLKcat and UniKP for predicting the kinetic parameter *k*_*cat*_ (Li et al. 2022; Yu et al. 2023). These intelligent approaches facilitate the analysis of big data generated by multi-omics sequencing, and help to optimize the GEMs for a particular host strain. Nevertheless, experimental data are still the basis for training artificial intelligence models. More practical data feeds can ensure the reliability and availability of AI models. The future computational approaches could consider the comprehensive capacity of models towards different environmental factors (e.g. mining a regulatory factor that responds to multiplex stresses). AI is expected to drive advances in biology, especially in the design of robust microbial cell factories.

## Data Availability

Data sharing not applicable to this article as no datasets were generated or analyzed during the current study.
